# Thiol/Redox Metabolomic Profiling Implicates GSH Dysregulation in Early Experimental Graft versus Host Disease (GVHD)

**DOI:** 10.1371/journal.pone.0088868

**Published:** 2014-02-18

**Authors:** Jung H. Suh, Bindu Kanathezhath, Swapna Shenvi, Hua Guo, Alicia Zhou, Anureet Tiwana, Frans Kuypers, Bruce N. Ames, Mark C. Walters

**Affiliations:** 1 Children’s Hospital Oakland Research Institute, Oakland, California, United States of America; 2 Children’s Hospital and Research Center Oakland, Oakland, California, United States of America; 3 Division of Blood and Marrow Transplantation, Children’s Hospital and Research Center Oakland, Oakland, California, United States of America; 4 Department of Pathology, Children’s Hospital and Research Center Oakland, Oakland, California, United States of America; Instituto de Biociencias - Universidade de São Paulo, Brazil

## Abstract

Graft-versus-host disease (GVHD) is a common complication of allogeneic bone marrow transplantation (BMT). Upregulation of inflammatory cytokines precedes the clinical presentation of GVHD and predicts its severity. In this report, thiol/redox metabolomics was used to identify metabolic perturbations associated with early preclinical (Day+4) and clinical (Day+10) stages of GVHD by comparing effects in Syngeneic (Syn; major histocompatibility complex- identical) and allogeneic transplant recipients (Allo BMT) in experimental models. While most metabolic changes were similar in both groups, plasma glutathione (GSH) was significantly decreased, and GSH disulfide (GSSG) was increased after allogeneic compared to syngeneic recipient and non-transplant controls. The early oxidation of the plasma GSH/GSSG redox couple was also observed irrespective of radiation conditioning treatment and was accompanied by significant rise in hepatic protein oxidative damage and ROS generation. Despite a significant rise in oxidative stress, compensatory increase in hepatic GSH synthesis was absent following Allo BMT. Early shifts in hepatic oxidative stress and plasma GSH loss preceded a statistically significant rise in TNF-α. To identify metabolomic biomarkers of hepatic GVHD injury, plasma metabolite concentrations analyzed at Day+10 were correlated with hepatic organ injury. GSSG (oxidized GSH) and β-alanine, were positively correlated, and plasma GSH cysteinylglycine, and branched chain amino acids were inversely correlated with hepatic injury. Although changes in plasma concentrations of cysteine, cystathionine (GSH precursors) and cysteinylglycine (a GSH catabolite) were not significant by univariate analysis, principal component analysis (PCA) indicated that accumulation of these metabolites after Allo BMT contributed significantly to early GVHD in contrast to Syn BMT. In conclusion, thiol/redox metabolomic profiling implicates that early dysregulation of host hepatic GSH metabolism and oxidative stress in sub-clinical GVHD before elevated TNF-α levels is associated with GVHD pathogenesis. Future studies will probe the mechanisms for these changes and examine the potential of antioxidant intervention strategies to modulate GVHD.

## Introduction

Graft-versus-host disease (GVHD) is an important complication of allogeneic hematopoietic stem cell transplantation (HSCT), and it limits the wider application of this curative treatment option [1], [2]. The pathogenesis of GVHD classically occurs in 4 distinct phases: 1) a first phase initiated by tissue injury that accompanies pre-transplant conditioning, 2) employment of host antigen presenting cells (APC) during an activation phase, 3) a donor T cell activation phase culminating in a cytokine storm, and 4) an effector phase during which activated effector T cells, natural killer (NK) cells, macrophages, and cytokines cause end-organ damage [1], [2]. Inflammatory cytokines, such as IL-1 [3], IL-2 [4], TNF-α [4] and IFN-γ [5], are elevated after allogeneic HSCT and perpetuate GVHD through direct cytotoxic effects on host tissues and by priming and activating immune effector cells [6]. The immunological mediators of GVHD have been investigated extensively, however biochemical and sub-cellular changes that precede and are mechanistically linked to T cell activation and cytokine dysregulation are not well characterized.

Oxidative stress is an unavoidable consequence of HSCT and may be an important exacerbating factor in GVHD. Owing to the contributions of pre-existing disease conditions and the requirement for conditioning regimens that increase cellular reactive oxygen species (ROS), oxidative stress is elevated in all HSCT recipients [1], [2], [7], [8]. Oxidatively modified membrane lipids, proteins and nucleic acids are known ligands for innate immune cell activation. Triggering damage-associated molecular pattern (DAMP) receptors may facilitate alloantigen presentation and donor T-cell activation required for GVHD initiation [1], [2]. Conditions that increase oxidative stress, such as iron-overload, are associated with increased risk for complications of HSCT, including GVHD [3], [9], [10]. Furthermore, in a study of Allo BMT recipients, there was a significant correlation between urinary F_2_-isoprostanes (an *in vivo* biomarker of lipid oxidation) and activation status of nuclear factor-kappa B (NF_k_B), a key transcription factor controlling the expression of inflammatory mediators and cytokines [4], [11], [12].

Allogeneic BMT is associated with increased oxidative stress during the active effector phase of GVHD [4], [5], [13]–[16]. Excess NO production was previously observed in both clinical GVHD [4], [13], [14] and experimental models [5], [15], [16]. Interestingly, two case studies reported that increases in serum nitrate/nitrite concentration indicative of inducible nitric oxide synthase activation, preceded clinical onset of GVHD [13], [14]. Alloantigen-activated T cells exhibit higher cellular mitochondrial ROS generation and contain less glutathione (GSH) than their syngeneic counterparts [6], [17]. Alloreactive T cells also induce epithelial genomic instability through generation of oxidative stress *in vitro*, which could explain why GVHD is associated with increased epithelial genomic instability in patients [18].

In addition to increased ROS generation, impaired antioxidant defense capacity following Allo BMT could also contribute to oxidative stress. Glutathione (GSH) is an endogenously synthesized sulfur amino acid (SAA)-containing tripeptide, which plays a principal role in cellular redox regulation. GSH synthesis is coordinately regulated through four sequentially interconnected pathways: transmethylation (TM); transsulfuration (TS); glutathione synthesis (GS); and glutathione recycling (GR) [5], [19]. These pathways generate homocysteine (Hcy), cysteine (Cys), GSH, and cysteinylglycine (Cysgly), all of which have labile sulfhydryl groups. Cys and GSH are the two most abundant plasma SAA compounds and are reversibly oxidized and reduced in cells by NADPH-driven processes [6], [20]. Thus, quantification of Cys/CySS and GSH/GSSG redox potentials provides accurate measures of balance between oxidative and anti-oxidative processes in biological systems [20], [21].

The redox state of the GSH/GSSG redox couple is normally tightly regulated (±7%) but it decreases in response to tissue injury, inflammation, and exposures to toxicants [20]. For example, in humans, plasma GSH/GSSG redox potentials decline by ∼15–20% following chemotherapy, and also in smokers, and patients suffering from diabetes and sepsis [20], [22]. In mouse models, similar oxidation of plasma GSH occurs during acute endotoxin-mediated lung injury [22], [23]. Independent of their cellular antioxidant effects, altered extracellular GSH oxidation states have been shown to enhance expression of adhesion molecules (VCAM, ICAM), mitochondrial and NADPH oxidase–dependent ROS generation, and IL-1B-mediated inflammatory signaling [24]–[26].

Although the importance of GSH in modulating inflammation has been established, it is not clear how GSH metabolism changes early after GVHD initiation and might mediate tissue injury. Clinical studies have shown that plasma and erythrocyte GSH and antioxidant enzymes activities decline after HSCT [7], [8], but how the plasma antioxidant defense system might relate to GVHD progression is not known. In experimental rodent models, pulmonary and hepatic GSH loss was observed in Allo BMT models of idiopathic pneumonia syndrome (IPS) [27]. A more recent study of experimental GVHD reported that GVHD increases erythrocyte oxidant generation and intracellular GSH relative to normal controls [28]. However, the absence of Syn controls, and single time point design together makes it difficult to ascertain the role of GSH perturbation in early GVHD pathogenesis.

Given the multidimensional roles of SAA and other amino acid metabolites in processes directly related to GVHD pathology, we hypothesized that comprehensive profiling of these compounds during the development of GVHD would provide new mechanistic insights into its complex pathobiology, particularly in its early phases. In this report, a thiol/redox metabolomics assay [29], [30] was used to simultaneously quantify redox states of SAAs and other amino acid metabolites in plasma and target organs during the development of experimental GVHD. These SAA-derived metabolites have an important regulatory function during inflammation by acting as ROS-scavenging antioxidants and by modulating redox states of protein thiols [3], [25], [31]. Depletion of tissue and systemic SAA-derived metabolites may promote cellular injury and apoptosis and also trigger a series of events that up-regulate inflammatory pathways [4], [32]. The possible involvement of SAA redox metabolism in GVHD has not been investigated, but the important role that it plays in early inflammatory signaling in other disease models [4], [32]–[34] suggests that it could play a critical role in the early stages of GVHD.

Our results demonstrate that oxidation of the host GSH-regulated redox system and failure of compensatory upregulation of GSH-synthesis enzymes occur prior to any evidence of change in amino acids known to be sensitive to inflammation. These results also indicate that this early shift in redox regulation precedes GVHD initiation as established by rise in circulating TNF-α. Lastly, the utility of plasma metabolomics to identify biomarkers of hepatic injury during the early clinical phase of GVHD is demonstrated.

## Materials and Methods

### Ethics Statement

All procedures were performed in compliance with the recommendations in the *Guide* and the *US Government Principles for the Utilization and Care of Vertebrate Animals Used in Testing, Research, and Training*. The protocol was approved by the CHORI Institutional Animal Care and Use Committee (IACUC Assurance No: A3631-01). All invasive procedures were performed under isofurane anesthesia, and efforts were made to minimize suffering at all times. Sacrifice procedures were performed in the morning following overnight fasting. Animals were sacrificed in their home cage by CO_2_ inhalation to effect and followed by exsanguination by cardiac puncture to obtain blood. All other tissues were harvested in sterile hood following cardiac puncture.

### Mice

Female mice C57BL/6 (B6: H-2^b^/CD45.2^+^, Thy1.2), BALB/c (H-2d/CD45.2^+^), B6D2F1 (H-2^bxd^, CD45.2^+^, Thy1.2^+^), and B6.PL-Thy1a (B6. Thy1.1: H-2^b^, CD45.2^+^, Thy1.1^+^) were purchased from The Jackson Laboratories (Bar Harbor, ME, USA) and/or inbred at the animal facility of Children’s Hospital Oakland Research Institute (CHORI) (Oakland, CA, USA). All animals were 8–12 weeks of age at the time of transplantation. Following transplantation, animals were fed standard fat-chow and maintained in micro-isolator cages in pathogen free environment. Animals were given Pen/Strep antibiotic water at a dose of 100 units/ml following lethal-radiation.

### Bone Marrow Transplantation and GVHD Assessment

Mice underwent transplantation in accordance with the protocol described previously [17], [35], [36]. Briefly, recipient mice received lethal (1100 cGy) x-ray irradiation using RS-2000 x-ray biological irradiator (160 kV, 4.2 kW, radiation dose of 96 rads/mt) (RadSource Technologies, Inc., Alpharetta, GA, USA). Radiation was administered as two fractions, 4 hours apart, to minimize gastrointestinal toxicity. T cell-depleted (TCD) bone marrow (BM) cells (10×10^6^) plus either CD90^+^ (5×10^6^) or CD4^+^ (3×10^6^) T cells from respective allogeneic (BALB/c) or syngeneic (B6. Thy1.1) donors were injected intravenously into recipient animals on day 0. To induce GVHD in unirradiated host, 50×10^6^ whole splenocytes from B6 Thy1.1 donor mice were infused into allogeneic (B6D2F1) and syngeneic (B6) mice. Survival was monitored daily, and body weights and GVHD clinical scores of recipients were measured thrice weekly, up to 30 days post-transplantation and then weekly for 6 months. The degree of systemic acute GVHD was assessed by a scoring system that incorporates 5 clinical parameters–weight loss, posture (hunching), activity, fur texture, and skin integrity, as described previously [37]. The mice exhibiting signs of severe GVHD (>6) were euthanized and the gut, liver, lungs and skin were harvested.

### Histologic Analysis

Representative samples from the liver, gut and lungs were placed in 10% phosphate buffered formalin, embedded in paraffin, cut into 5 µm sections and stained with hematoxylin and eosin for histopathological examination. A pathologist reviewed histopathology sections in a blinded manner to assess for GVHD. A semi-quantitative system of scoring was used as previously described [5], [19], [37]. This scoring evaluated apoptosis, portal infiltrates, lobular infiltrates, bile duct damage and vascular endothelialitis. The scoring system for each of these parameters denoted 0 as normal, 1 as mild, 2 as moderate and 3 as severe. The percentage of mice suffering from moderate to severe GVHD at the time of euthanasia was noted.

### Cytokine Analysis

Plasma samples from transplanted animals were aliquoted and batched analysis was performed using FlowCytomix bead based immunoassay (eBioscience, San Diego, CA) in 96 well microplate format, in accordance with manufacturer’s instructions. The standard curves were created using 5-fold dilution of appropriate standard in culture medium and serum samples were analyzed using mouse Th1/Th2 10 plex kit flowcytomix.

### Protein Carbonyl Analysis

Protein carbonyls were measured as a biomarker of hepatic protein oxidation by using the OxiSelect™ Protein Carbonyl ELISA Kit (Cell Biolabs Inc, San Diego, CA). Briefly, flash-frozen liver tissues (25 mg) were homogenized in 1 ml of phosphate-buffered saline solution by using the Fastprep FP120 centrifuge homogenizer (Qbiogene, Inc, Carlsbad, CA). Homogenized samples were subsequently centrifuged at 20,000 x g to obtain cytosolic protein fraction. Protein carbonyls in hepatic cytosolic fraction (100 µg protein/ml) were quantified by dinitrophenylhydrazine (DNPH) derivatization followed by specific detection by anti-DNP antibody in accordance with manufacturer’s instructions.

### Fluorescence Activated Cell Sorter (FACS) Analysis

Hepatocytes were stained with CellROX™ (5 µM) and Thiol Tracker Violet (TTV; 20 µM) (Invitrogen) per manufacturers instructions. FACS analysis was performed using LSR Fortessa (BD Biosciences). Data was analyzed using Flowjo software (Treestar, Ashland, OR).

### Redox Metabolomics

Plasma and hepatic SAA and other amino acid metabolites were quantified using a liquid chromatography linked tandem mass-spectrometry (LC/MS/MS) assay [6], [20], [29], [30].

### Redox Calculation

The plasma redox potential for glutathione (GSH) was calculated using the Nernst equation [20], [21]; Eh = (redox potential at pH 7.4) +30 log ([oxidized disulfide]/[reduced thiol]^2^). The standard redox potential used was −264 mV.

### Quantitative Real Time PCR of Phase II Genes

A portion of each liver was excised, flash frozen in liquid nitrogen, stored at −80°C and homogenized using a Fast Prep system (MP Biomedicals). Total RNA was isolated from mouse livers using an RNeasy Mini Kit (Qiagen, Valencia, CA). cDNA was prepared from 1 µg of total RNA per group using QuantiTect Reverse Transcription Kit (Qiagen). Relative transcript amounts of γ-Glutamylcysteine Ligase Catalytic Subunit (GCLC) was quantified using the [delta][delta] Ct method with 18S rRNA as a control. PCR reactions were carried out using the ABI 7900 Real Time qPCR system (Life Technologies, Fredrick, MD) under following cycling conditions: 95°C for 10 mins, and 40 cycles at 95°C for 10 mins and 60°C for 1 min. The primers used for GCLC were: FP 5′CCACTGAGCTGGGAAGAGAC-3′ and RP 5′-TCATGATCGAAGGACACCAA-3′ and for 18S ribosomal RNA were: FP5′-GTAACCCGTTGAACCCCATT-3′ and RP 5′-CCATCCAATCGGTAGTAGCG-3′.

### Survival, Clinical GVHD Score and Serum Cytokine Analysis

Survival data in each group were generated using Kaplan–Meier lifetime survival probability methodology and the log-rank (Mantel–Cox) test. GVHD scores and serum cytokine concentrations were analyzed by ANOVA with Tukey’s Honest Significance Test (HSD) post-hoc tests. PRISM software (SAS Institute, Cary, NC, USA) was used for these tests and a *p* value <0.05 was considered statistically significant.

### Statistical Analysis of Metabolomic Data

All of the metabolomic statistical tests described below were performed using the Metaboanalyst software platform [20], [38]. Metabolite concentrations were normalized by the median concentration quantified from untreated control samples (N = 5). Normalized values were cube root transformed, Pareto scaled and mean-centered prior to statistical testing.

### Univariate Analysis

For univariate analysis of data obtained from baseline controls, Syn, Allo BMT at Day+4 and +10 time points, statistical analysis of microarray (SAM) was performed with Delta values adjusted to minimize false discovery rate below 10%. Tukey’s Honestly Significant (Tukey’s HSD) was used to further quantify the differences between the groups.

### Principal Component Analysis (PCA)

PCA is an unsupervised classification method that projects high-density data to new coordinated system with much smaller sets of variables (principal components; PC), which describe the variability in the data. Each principal component is orthogonal to each other and each PC explains the greatest source of variance remaining after previous PCs have been established. PCA produces two plots; 1) scores plot: projects each sample on a new coordinate system composed of PCs and are used to reveal the intrinsic structure of data set in terms of variance, 2) loading plot: displays variables that contributes to the group separation.

### Partial-Least-Squares Discriminant Analysis (PLS-DA)

PLS-DA is a supervised technique where the discriminant model is built with a prior knowledge of the group membership of set of samples and is created to best explain the group memberships of individual samples. PLS-DA has two outputs: 1) score plot shows the sample distribution in the coordinate system composed to the components selected, 2) Variable importance in Projection (VIP) values which is a computation of influence of every x term in the model on the group classification and larger VIP values indicate a greater influence of x on group discrimination and generally, a VIP value of ≥ 1 are considered significant. Because PLS-DA is prone to over-fitting errors, the significance of group discrimination by multivariate models was cross-validated with the use of “leave-one-out” and permutation testing.

### Hierarchical Clustering Analysis (HCA) and Heat-map Generation

Variables that were found to contribute most to the group discrimination were used for hierarchical clustering analysis (HCA) using Pearson’s test for distance measures and Ward’s minimum variance method was used for clustering. Samples are in rows and variables are in columns. The colors vary from deep blue to dark brown to indicate data values change from very low to extremely high.

## Results

### Establishment of the GVHD Time Course

**Figure 1:**
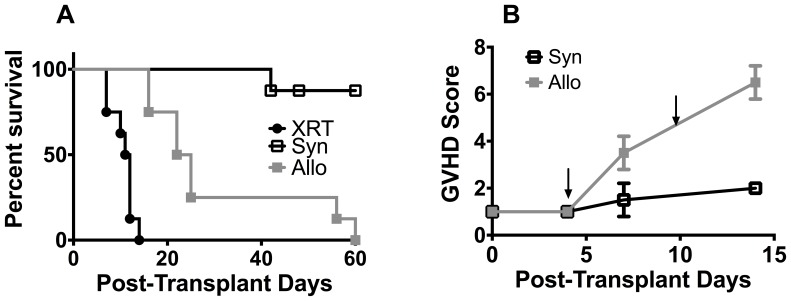
Survival and clinical course following major histocompatibility complex mismatched bone marrow transplantation (BMT). A) Survival analysis of C57BL/6 mice following lethal radiation (XRT) alone, allogeneic (Allo) BMT (BALB/c ^(H-2d/CD45.2+)^→ C57BL/6 ^(Thy1.2: H-2b/CD45.2+)^) and syngeneic (Syn) BMT (C57BL/6 ^(Thy1.1: H-2b/CD45.2+)^→ C57BL/6 ^(Thy1.2: H-2b/CD45.2+)^). B) A time course of GVHD score changes in Allo and Syn BMT mice. Based on this time course, we chose post-transplant Day+4 (pre-clinical) and Day +10 (clinical onset) time-points (arrows) for metabolomic analysis in subsequent experiments.

GVHD was induced in the fully MHC-mismatched model consisting of BALB/c → B6 following lethal irradiation (**Table 1**, Model 1). The survival time course (**Figure 1**; Panel A) monitored over 60 days shows that mice receiving the lethal radiation conditioning regimen alone (XRT; N = 8) had a median survival of 11.5 days (100% mortality at 18 days), while 87.5% of Syn BMT recipients (Syn; N = 8) survived during the first 60 days following transplantation. Allo BMT recipients (N = 8) had a median survival of 23.5 days. The clinical GVHD score in the Allo group started to increase significantly at post-transplant Day 7 (**Figure 1**, Panel B). Based on this time course, post-transplant Day+4 (pre-clinical) and Day +10 (clinical GVHD expressed) time points were chosen for metabolomic analysis.

**Table 1:** Description of Experimental BMT Model.

	Donor Strain	Recipient Strain	Mismatch Type	Conditioning Regimen	Major T-cell Type	Cell Type & Dosage
**Model 1. GVHD with Conditioning Regimen**						
**Syngeneic** **(Syn)**	C57/Bl6(Thy1.1: H-2b)	C57/Bl6(Thy1.2: H-2b/CD45.2+)		1100 cGy	CD4+ and CD8+	TCD-BM cells (10×106) with CD4+
**Allogeneic** **(Allo)**	Balb/C(H-2d/CD45.2+)	C57/Bl6(Thy1.2: H-2b/CD45.2+)	MHC-I, -II,and miHAs	1100 cGy	CD4+ and CD8+	TCD-BM cells (10×106) with CD4+
**Model 2. GVHD without Conditioning Regimen**						
**Syngeneic** **(Syn)**	C57/Bl6(Thy1.1: H-2b/CD45.2+)	C57/Bl6(Thy1.2: H-2b/CD45.2+)		None		Whole Splenocytes (50×106 cells)
**Allogeneic** **(Allo)**	C57/Bl6(Thy1.1: H-2b/CD45.2+)	B6D2F1(H-2bxd, CD45.2+,Thy1.2+)	MHC-I, -II,and miHAs	None	CD4+ and CD8+	Whole Splenocytes (50×106 cells)

MHC – Major histocompatibility complex.

MiHAs – Minor histocompatibility antigens.

### Amino Acid Metabolite Profiles Following Allo Transplantation Indicate Early Failure of the GSH Antioxidant Defense System

Plasma amino acid metabolomic changes following Syn and Allo BMT at post-transplantation Days +4 and +10 were compared to baseline wild-type (WT) controls. Of the 40 metabolites measured, 16 metabolites changed significantly compared to baseline and are shown in **Table 2**. The most striking change occurred at Day+4 in the GSH concentrations where the Syn mice maintained adequate levels even after lethal irradiation, but levels were severely depleted in the Allo mice. This result suggests that the antioxidant response mechanism is impaired in Allo but not Syn animals during this early phase. Consequences of these changes were reflected in the more oxidized GSH/GSSG redox potential (more positive = more oxidized) (**Table 2** **)**.

**Table 2:** Change in metabolite concentrations following allogeneic and syngeneic bone marrow transplantation.

Metabolite	Control	SyngeneicDay+4	SyngeneicDay +10	AllogeneicDay+4	AllogeneicDay+10	p	FDR^6^
***Metabolites that are differentially changed in Allo versus Syn BMT***							
**F.GSH** 1	47.3±4.6^a^	41.7±11.4^a^	42.9±1.2^a^	15.2±4.6^b^	15.6±4.0^b^	0.001	0.005
**GSSG** 2	3.4±1.7^a^	5.7±0.6^a^	5.3±1.5^a^	18.9±8^b^	8.1±6.7^a^	0.000	0.003
**GSH/GSSG (mV)** 3	−148.0±5.3^a^	−158.0±6.7^b^	−160.6±2.8^b^	−116.7±5.8^c^	−128.9±11.3^a,c^	0.000	0.000
***Metabolites that are differentially changed relative to baseline control in Allo and in Syn BMT***							
**Ergothioneine**	0.3±0.2^a^	6.2±1.5^b^	8.7±1.1^b^	5.6±1.0^b^	8.2±3.6^b^	0.000	0.000
**T. Homocysteine** 4	6.3±3.2^a^	19.2±6.4^b^	6.9±3.4^a^	17.8±5.7^b^	10.8±2.1^a^	0.001	0.005
**Phenylalanine**	38.9±15.1^a^	100.8±30.7^b^	147.7±23.9^b^	87.0±17.3^a,b^	98.7±38.2^b^	0.001	0.005
**Tryptophan**	73.7±21.0^a^	107.6±26.8^a^	159.3±38.6^b^	123.7±26.4^a^	160.2±24.5^b^	0.001	0.006
**Glutamate**	41.3±5.0^a^	51.7±18.2^a^	107.1±22.7^b^	48.7±15.2^a^	100.2±37.7^b^	0.001	0.006
**Isoleucine**	86.5±48.8^a^	248.3±89.7^b^	263.6±1.0^b^	219.2±27.4^b^	225.2±90.3^b^	0.003	0.011
**Beta-alanine**	1.1±0.8^a^	1.9±0.3^a^	3.4±0.2^c^	1.8±0.4^a^	2.6±0.8^b^	0.009	0.026
**Citrulline**	55.1±22.2^a^	17.4±7.3^b^	10.3±5.6^b^	16.2±7.5^b^	31.9±32.4^b^	0.009	0.026
**Aspartate**	3.0±0.7^a^	1.8±0.4^b^	2.4±0.1^a,b^	1.9±0.2^b^	2.0±0.5^b^	0.010	0.029
**Histidine**	62.9±27.0^a^	33.9±7.9^b^	38.3±10.3^b^	28.3±5.2^b^	39.2±9.7^b^	0.019	0.049
**Ornithine**	43.5±12.5^a^	19.8±8.7^b^	40.9±10.5^a^	30.5±13.3^a,b^	41.34±13.5^b^	0.026	0.063
**Valine**	112.1±24.9^a^	130.4±40.3^a^	234.1±26.3^b^	151.1±41.2^a,b^	160.5±67.8^a,b^	0.037	0.079
**Tyrosine**	58.5±30.5^a^	113.5±32.9^b^	102.5±7.5^a,b^	101.6±19.6^a,b^	80.1±23.5^a,b^	0.035	0.079
**T. Cysteine** 5	167.5±57.6^a^	233.0±92.1^a,b^	251.7±52.7^a,b^	223.4±63.0^a,b^	305.3±14.4^b^	0.049	0.098

Lethally irradiated B6 recipients (Irradiated) were transplanted with 5×10^6^ T-cell depleted bone marrow cells (TCD-BM) and 3×10^6^ CD90^+^ T-cells from B6 Thy1.1 (Syngeneic) or Balb/C (Allogeneic) donor mice.

All units are µmol/L unless otherwise noted and values are expressed as Mean±SD.

Statistical Analysis of Microarray with Tukey’s post hoc test was used to determine statistical differences between groups.

Different letters denote significant differences among treatment groups (e.g. ^a^ versus ^b^).

1 F. GSH - non-protein bound Free GSH.

2 GSSG – glutathione disulfide.

3 GSH/GSSG (mV) – GSH/GSSG redox potential was calculated using the Nernst Equation as described in methods.

4 T. Homocysteine – Total Homocysteine concentration obtained following plasma reduction with dithiothreitol (DTT).

5 Cysteine – Total Cysteine concentration obtained following plasma reduction with dithiothreitol (DTT).

6 FDR- False discovery rate.

The liver is the major site for GSH synthesis and is also a target organ of GVHD [20], [22], [39]. As in the plasma, GSH was the only metabolite in liver homogenates in which the concentration significantly decreased in Allo BMT mice compared to Syn mice at Day+4 (**Table 3**). As shown, hepatic total GSH in Syn BMT was initially elevated at Day +4 and subsequently decreased to baseline WT values. In contrast, the hepatic GSH did not increase in Allo BMT mice relative to baseline at Day +4 (**Table 3**). By Day+10, the liver GSH concentration had decreased to values significantly lower than baseline (**Table 3**). These results suggest that failure of Allo BMT hosts to increase hepatic GSH synthesis during early GVHD is the cause of the depletion of plasma GSH and a higher GSSG level.

**Table 3:** Metabolomic Changes in the liver following syngeneic and allogeneic bone marrow transplantation.

Metabolite	Control	SyngeneicDay+4	SyngeneicDay +10	AllogeneicDay+4	AllogeneicDay+10	p	FDR^4^
***Metabolites that are differentially changed in Allo versus Syn BMT***							
**T.GSH** 1	40.3±14.1^a^	68.0±16.3^b^	32.3±4.9^a^	43.2±12.6^a^	25.6±6.8^c^	1.2E-02	1.8E-02
**F.GSH** 2	25.9±5.6^a^	28.1±12.8^a^	12.9±3.0^a,b^	29.2±11.6^a^	4.9±4.0^b^	4.9E-04	1.1E-03
**GSH/GSSG ratio**	25.4±5.6^a^	9.6±8.9^b^	1.7±0.6^b^	3.8±1.6^b^	0.5±0.5^c^	1.1E-04	3.3E-04
**AdoHcy**	5.8±2.4^a^	0.1±0.04^b^	0.1±0.02^b^	0.2±0.1^c^	0.7±0.03^d^	3.5E-09	1.5E-07
***Metabolites that are differentially changed relative to baseline control in Allo and in Syn BMT***							
**AdoMet**	8.4±7.4^a^	4.6±2.7^a,c^	0.3±0.04^b^	2.5±1.1^a,c,d^	0.1±0.1^b^	1.2E-08	2.7E-07
**Thiaproline**	0.03±0.02^a^	0.7±0.3^b^	0.6±0.2^b^	0.6±0.2^b^	0.6±0.1^b^	1.2E-07	1.7E-06
**α-aminoadipate**	0.5±0.3^a^	4.7±1.7^b^	0.4±0.2^a^	4.3±2.5^b^	0.3±0.1^a^	4.9E-07	5.4E-06
**Aspartate**	3.7±2.5^a^	29.8±10.8^b^	31.0±15.0^b^	22.7±7.0^b^	28.3±2.7^b^	7.8E-06	6.8E-05
**Cystine**	0.03±0.0^a^	0.2±0.1^b^	0.08±0.05^a^	0.3±0.2^b^	0.1±0.0^a^	1.3E-05	7.6E-05
**Ophthalmate**	0.04±0.0^a^	0.6±0.2^b^	0.5±0.^4a,b^	0.7±0.2^b^	0.4±0.4^a,b^	1.4E-05	7.6E-05
**Methionine**	2.1±0.5^a^	3.2±0.8^a^	6.0±1.5^b^	3.9±0.6^a^	6.1±1.2^b^	2.1E-05	9.3E-05
**3-methylhistidine**	0.02±0.00^a^	0.2±0.1^b^	0.26±0.1^b^	0.3±0.2^b^	0.3±0.1^b^	2.1E-05	9.3E-05
**Cys/CySS ratio**	13.0±3.1^a^	5.1±2.8^b^	2.5±1.4^b^	4.8±3.3^b^	1.0±0.4^b^	2.8E-05	1.1E-04
**T.Homocysteine** 3	0.3±0.1^a^	2.5±1.6^b^	0.3±0.0^a^	4.1±2.7^b^	0.3±0.1^a^	2.9E-05	1.1E-04
**Proline**	2.8±1.5^a^	14.9±5.5^b^	21.6±7.1^b^	14.2±2.4^b^	21.1±3.6^b^	3.3E-05	1.1E-04
**Glutamine**	10.2±9.2^a^	42.0±13.9^b^	29.0±1.4^a,b^	39.8±7.0^b^	30.8±13.7^b^	4.7E-05	1.5E-04
**Cysteine**	0.4±0.1^a^	2.3±1.2^b^	0.3±−0.04^a^	3.5±4.2^b^	0.2±0.1^a^	3.1E-04	8.4E-04
**Serine**	12.8±6.7^a^	32.9±10.0^b^	53.6±9.4^b^	41.7±5.7^b^	54.3±8.7^b^	3.7E-04	9.5E-04
**Spermine**	19.9±7.2^a^	7.2±1.1^b^	6.0±2.4^b^	10.0±3.3^b^	8.9±3.7^b^	4.3E-04	1.1E-03
**Asparagine**	5.7±2.5^a^	16.6±6.0^b^	19.5±5.5^b^	20.5±2.8^b^	22.1±5.4^b^	5.9E-04	1.2E-03
**Ergothioneine**	0.1±0.1^a^	1.28±0.5^a,c^	3.1±0.0^b,c^	3.0±2.5^b,c^	5.0±0.4^b^	5.5E-04	1.2E-03
**Alanine**	32.8±14.9^a^	75.2±25.3^b^	81.5±15.8^b^	82.2±9.7^b^	74.2±11.2^b^	1.4E-03	2.8E-03
**Threonine**	6.0±2.6^a^	2.5±0.6^a,c^	14.2±6.5^a,b^	8.5±6.2^a,c,d^	17.9±4.9^b,d^	1.6E-03	3.0E-03
**Glutamate**	25.4±9.8^a^	78.5±29^b^	68.1±37.1^b^	66.3±15.7^b^	59.4±11.7^b^	2.3E-03	4.3E-03
**GSSG^5^**	1.0±0.2^a^	2.5±1.6^a,b^	4.2±2.4^a,b^	4.2±2.7^a,b^	5.8±2.0^b^	5.0E-03	8.8E-03
**Ornithine**	6.3±1.5^a^	15.2±6.0^b^	14.2±4.2^b^	12.9±2.5^b^	15.4±3.8^b^	6.1E-03	1.0E-02
**Valine**	8.6±0.8^a^	17.4±7.0^b^	16.6±3.0^a,b^	14.6±3.9^a,b^	16.9±2.1^b^	1.1E-02	1.7E-02
**Arginine**	0.1±0.0^a^	1.2±0.4^b^	0.2±0.2^a^	0.8±0.3^b^	1.1±0.5^b^	1.4E-02	2.1E-02
**Tryptophan**	3.4±1.7^a^	2.5±0.5^a,b^	1.6±0.4^b^	1.9±1.0^a,b^	1.5±0.3^b^	1.5E-02	2.2E-02
**Phenylalanine**	6.4±1.6^a^	13.8±4.5^b^	11.4±1.7^a,b^	10.5±3.8^a,b^	10.9±2.1^a,b^	3.3E-02	4.3E-02
**Lysine**	16.9±3.3^a^	35.6±15.4^a,b^	31.9±10.6^a,b^	25.2±5.3^a,b^	33.9±7.4^b^	3.3E-02	4.3E-02
**Sarcosine**	0.4±0.3^a^	1.0±0.5^b^	0.8±0.7^a,b^	0.8±0.3^a,b^	0.6±0.4^a,b^	4.0E-02	5.0E-02

Lethally irradiated B6 recipients (Irradiated) were transplanted with 5×10^6^ T-cell depleted bone marrow cells (TCD-BM) and 3×10^6^ CD90^+^ T-cells from B6 Thy1.1 (Syngeneic) or Balb/C (Allogeneic) donor mice.

All units are µmol/L unless otherwise noted and values are expressed as Mean±SD.

Statistical analysis of microarray with Tukey’s post hoc tests was used to determine statistical differences between the groups. Different letters denote significant differences among treatment groups (e.g. ^a^ versus ^b^).

1 T. GSH – Total GSH (non-protein bound Free GSH +2GSSG+GSH-mixed disulfides).

2 F. GSH - non-protein bound Free GSH.

3 T. Homocysteine – Total Homocysteine concentration obtained following plasma reduction with dithiothreitol (DTT).

4 FDR - False Discovery Rate.

To determine whether the observed decline in GSH is due to GVHD rather than to the conditioning regimen, GVHD was induced in unirradiated recipients using a paternal to F1 hybrid (B6 → B6D2F1) transplantation model (**Table 1**; Model 2). The B6 → B6D2F1 is a well-established model where lethal GVHD develops over the course of several months [22], [23], [40]. It has been noted that 33% of the B6D2F1 recipients challenged with B6 splenocytes develop bone marrow failure induced by acute GVHD. The remaining animals exhibited signs of subclinical GVHD with protracted immune system insufficiency [24]–[26], [41].

Comparison of plasma GSH and GSSG concentrations in controls, Syn and Allo BMT recipients at days +4 and +10 post T cell infusion showed significant alterations (**Table 4**). Despite the lack of radiation injury, adoptively transferred Allo splenocytes caused a significant decrease in plasma GSH concentration at Days +4 and +10 compared to both baseline controls and the Syn group. GSSG levels were significantly increased in Allo compared to Syn recipients, but only at Day+10. The plasma GSH/GSSG redox state as calculated by the Nernst equation showed a significant loss in antioxidant capacity at both Days +4 and +10 (**Table 4**). However, the extent of plasma GSH/GSSG redox potential loss was more modest than in the BALB/c →B6 model following lethal radiation (**Table 2**). Interestingly, analysis of hepatic GSH showed significant 40% increase in Syn mice while the levels in Allo did not change relative to baseline controls (**Table 4**). Hepatic GSSG levels in Allo mice were also significantly higher at Day+4. In the Syn group, hepatic GSH and GSSG levels had returned to baseline levels by Day+10 (**Table 4**). However, in the Allo group, hepatic GSH levels were significantly lower compared to Syn and to untreated controls (**Table 4**). These results suggest that a compensatory increase in hepatic GSH is impaired in the Allo group, irrespective of whether a conditioning regimen was applied.

**Table 4:** Plasma and liver GSH Changes following syngeneic and allogeneic bone marrow transplantation without conditioning regimen.

Metabolite	Control	SyngeneicDay+4	SyngeneicDay +10	AllogeneicDay+4	AllogeneicDay+10	p	FDR^4^
***Plasma GSH, GSSG and redox potential***							
**F.GSH** 1	47.3±4.6^a^	46.2±0.4^a^	39.4±5.8^a^	21.7±6.7^b^	21.4±10.7^b^	0.001	0.004
**GSSG** 2	3.4±1.7^a^	7.3±1.0^b^	7.7±3.5^b^	7.7±1.4^b^	14.9±11.3^c^	0.008	0.01
**GSH/GSSG (mV)** 3	−148.0±5.3^a^	−158.0±2.2^b^	−154.1±7.8^b^	−136.8±6.3^a,c^	−127.9±9.4^a,c^	0.04	0.05
***Liver GSH, GSSG and GSH/GSSG ratio***							
**F.GSH**	25.9±5.6^a^	36.3±4.5^b^	29.3±5.3^a^	24.7±19.5^a^	14.2±12.9^c^	0.001	0.006
**GSSG**	1.04±0.2^a^	4.5±3.7^b^	1.3±0.9^a^	12.2±9.9^b^	1.5±1.0^a^	0.001	0.006
**GSH/GSSG Ratio**	24.9±1.2^a^	8.2±0.7^b^	22.6±4.7^a^	2.1±4.3^c^	9.5±4.8^b^	0.003	0.011

GVHD was induced without conditioning in paternal in F1 hydrid (C57BL/6 → B6DBA2F1) model.

SAM analysis with Tukey’s HSD post hoc analysis was used to determine statistical differences among group.

All units are µmol/L unless otherwise noted and values are expressed as Mean±SD.

Different letters denote significant differences among treatment groups (e.g. ^a^ versus ^b^).

1 F.GSH - non-protein bound Free GSH.

2 GSSG – glutathione disulfide.

3 GSH/GSSG (mV) – GSH/GSSG redox potential was calculated using the Nernst Equation as described in methods.

4 FDR - False Discovery Rate.

### A Blunted GSH Antioxidant Defense System Response Involves Impaired Transcriptional Upregulation of γ-glutamylcysteine Ligase (GCL), the Rate-Limiting Enzyme in GSH Biosynthesis

**Figure 2:**
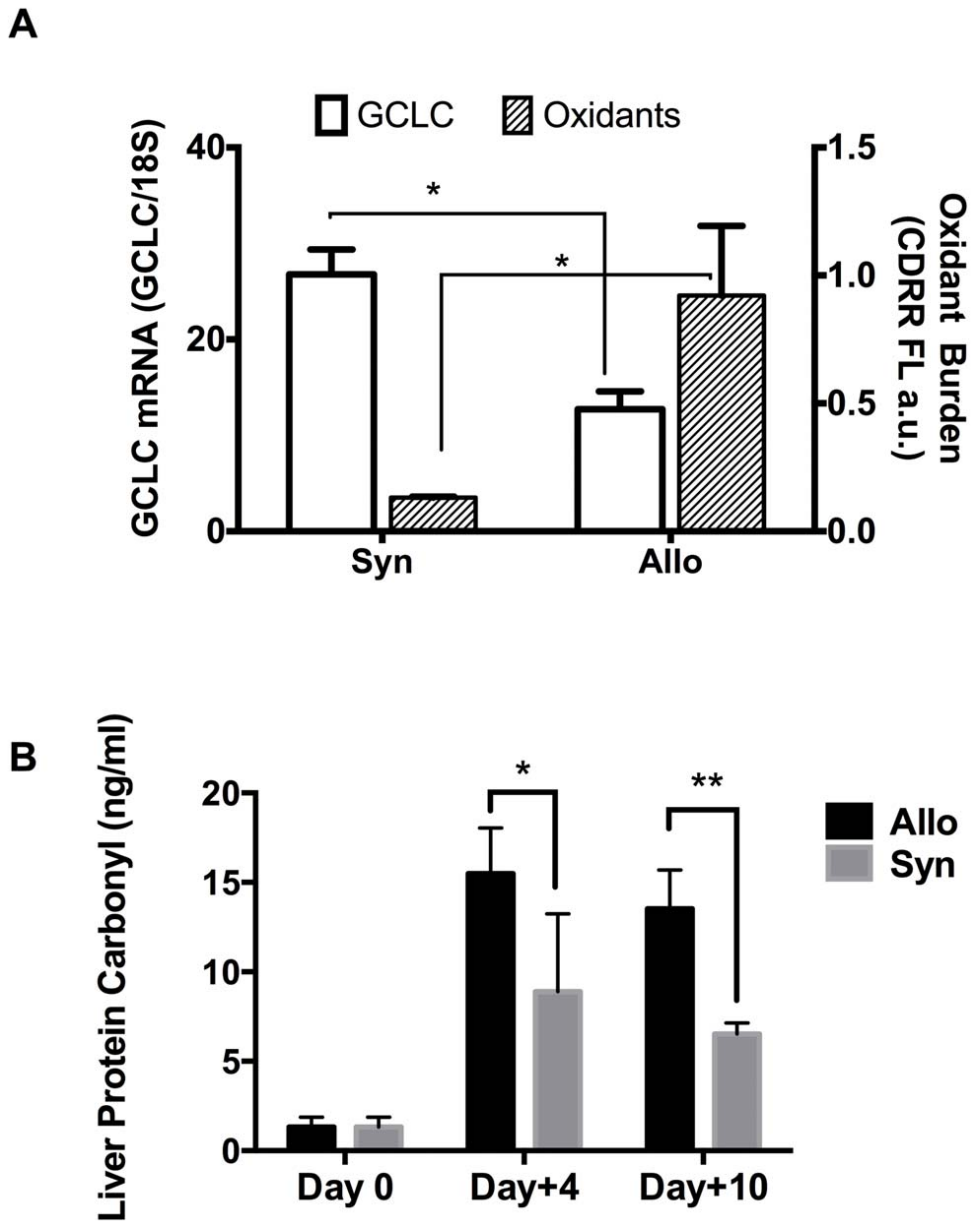
Decreased expression of the γ-glutamylcysteine ligase and increased hepatic oxidant generation and protein oxidative damage at Day+4 in Allo BMT. Panel A: At post-transplant day 4, hepatocytes from Syn (B6→B6) and Allo (Balb/C→B6) mice (N = 4 per group) were freshly isolated and the mRNA transcripta of the catalytic sub-unit of γ-glutamylcysteine ligase (GCLC; Green, left axis), were quantified by RT-PCR and normalized to 18S mRNA levels. Cellular reactive oxygen species (ROS) levels were estimated by flow-cytometric detection of CellRox Deep Red Reagent (CDRR) fluorescence. Results are Mean±SD. * = *p*<0.05. Panel B: Hepatic protein oxidative damage levels in non-transplanted controls (Day 0; N = 4), Syn (B6→B6; N = 3 per time point; Gray bar) and Allo (Balb/C→B6; N = 5 per time point; Black bar) BMT mice were measured by protein carbonyl ELISA assay as described in methods. Results are mean±SD. * = *p*<0.05 and ** = *p*<0.01.

Due to the central role of GSH in cellular antioxidant protective mechanisms, the induction of enzymes responsible for its synthesis represents a key adaptive response to oxidative injury. The synthesis of GSH from precursor amino acids requires γ-glutamylcysteine ligase (GCL) and GSH synthetase. GCL catalyzes the rate-limiting step. It is a heterodimer composed of catalytic (GCLC) and regulatory (GCLM) subunits. GCLC transcription is upregulated in response to increased oxidative stress or xenobiotic exposure. An inadequate GCLC transcriptional induction in response to a cellular ROS burden would result in oxidative stress. At post-transplant Day+4, hepatocytes from Allo BMT mice were freshly isolated and the cellular oxidant burden and relative abundance of GCLC mRNA were estimated by CellROX™ Deep Red reagent (CDRR) flow cytometric and RT-PCR assay, respectively. As shown in **Figure 2A**, cellular ROS levels increased by ∼4-fold at post-transplant Day+4 in Allo mice compared to the Syn BMT group, and the relative GCLC RNA transcript abundance was decreased by ∼50%. These results strongly suggest that transcriptional responses required for maintaining adequate cellular GSH levels, as occurred in the Syn BMT mice, are impaired early following Allo transplantation and before clinical GVHD develops.

Protein carbonyls are formed as a consequence of ROS-dependent protein oxidative modification and are stable biomarkers of oxidative stress. As shown in **Figure 2B**, hepatic protein carbonyl concentrations were significantly (p<0.05) higher in Allo BMT (15.5±2.5 ng/ml) as compared to both baseline controls (Day 0; 1.3±0.5 ng/ml) and Syn BMT (8.9±4.3 ng/ml) at Day +4 and was remained elevated at Day +10.

### Plasma and Hepatic Glutathione Depletion Precede Up-regulation of Inflammatory Cytokines

**Figure 3:**
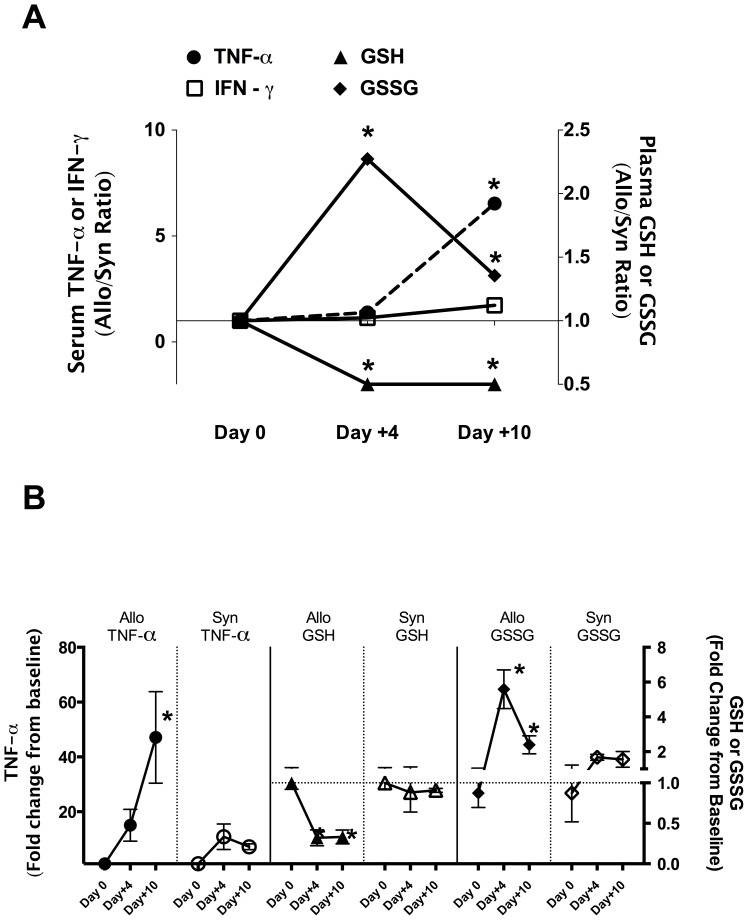
Temporal change in GSH oxidation and loss precedes the rise in TNF-α. Lethally irradiated B6 recipients (Irradiated) were transplanted with 5×10^6^ T-cell depleted bone marrow cells (TCD-BM) and 3×10^6^ CD90^+^ T-cells from B6 Thy1.1 (Syngeneic) or Balb/C (Allogeneic) donor mice (N = 4 per group). Serum TNF-α, plasma GSH and GSSG concentrations were normalized to average control values. **Panel A** shows the temporal patterns of mean Allo/Syn ratios of control-normalized serum TNF-α (left axis; circle dotted line), plasma GSH (open triangle, right y axis) and GSSG (open diamond right axis). **Panel B** shows the temporal changes in serum TNF-α, plasma GSH and GSSG for Allo and Syn groups separately and values are expressed as fold-change over baseline control mean. *Denotes significant differences between Allo and Syn BMT mice. Data represents mean ± SD.

To establish the temporal relationship between plasma GSH/GSSG redox potential change and inflammatory cytokine upregulation characteristic of GVHD, serum concentrations of IL-2, IFN-γ, and TNF-α were measured at post-transplant Days +4 and +10. Figure 3A shows the Allo/Syn ratios of baseline normalized values for the different end-points measured, whereas Figure 3B shows the fold-change from baseline values for Syn and Allo groups plotted separately for each of the endpoints measured. As shown in **Figure 3**, the control-normalized serum IFN-γ Allo/Syn ratio was not significantly different at the time points. Serum TNF-α concentrations were increased relative to baseline levels to a similar extent in both Syn and Allo mice at Day+4 (**Figure** **3**A and B). However, at post-transplant Day+10, serum TNF-α in Allo was significantly elevated relative to both non-transplant control and Syn mice (**Figure** **3**A and B). Divergent changes in plasma GSH and GSSG were observed at Day+4 post-transplant (**Figure** **3**A and B). Mean plasma GSH decreased by approximately 60% from baseline values in the Allo mice group at Day+4 and was maintained at this reduced level at Day+10 (**Figure** **3**B). In the Syn group, mean plasma GSH concentrations were maintained at levels similar to controls at both time points. Plasma GSSG concentrations in the Allo group increased by ∼6 fold at Day+4 and while it decreased by Day+10, the level was still significantly higher than in the Syn and non-transplant controls (**Figure** **3**A and B). In contrast, GSSG levels were only slightly elevated in the Syn group at Days +4 and +10. However, it should be noted that even with this small decline in GSSG, the plasma GSH/GSSG redox potential remained low (**Table 2**), primarily because plasma GSH is the major determinant of the GSH/GSSG redox potential. These data suggest that impaired GSH antioxidant defense compensation in Allo mice occurs before TNF-α upregulation at Day+10.

### Plasma GSH Depletion at Day+10 Correlates with Hepatic GVHD Severity

**Figure 4:**
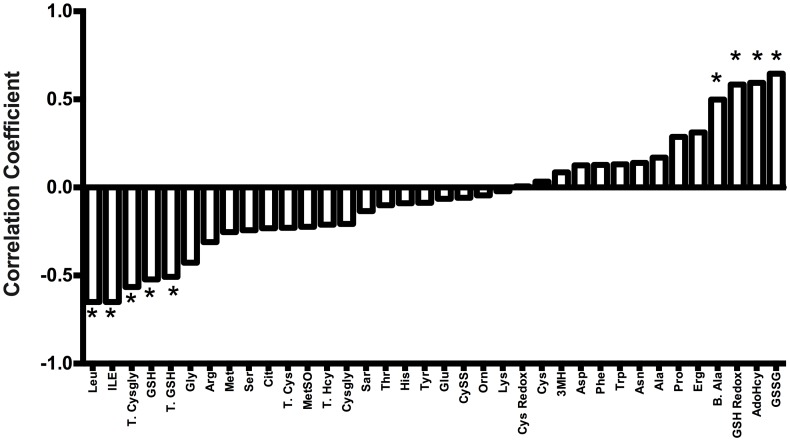
Significant correlations between hepatic GVHD scores and changes in plasma metabolite concentrations at Day +10. GVHD was induced without conditioning in paternal in the F1 hybrid (C57BL/6 → B6DBA2F1) model. Pearson correlation coefficient analysis was used to calculate the correlation between plasma metabolite concentrations and hepatic GVHD histopathological severity scoring obtained at Day+10. *Denotes metabolites that showed significant correlations to hepatic GVHD severity scores. Abbreviations: Leu – Leucine, Ile - Isoleucine, T. cysgly-Total cysteinylglycine, GSH-Free GSH, T.GSH- Total GSH, Gly-Glycine, Arg-Arginine, Met-Methionine, Ser-Serine, Cit-Citrulline, T.Cys-Total cysteine, MetSO-Methionine sulfoxide, T. Hcy-Total homocysteine, Cysgly-Cysteinylglycine, Sar-Sarcosine, Thr-Threonine, His-Histidine, Tyr-Tyrosine, Glu-Glutamate, CySS-Cystine, Orn-Ornithine, Lys-Lysine, Cys Redox-Cysteine redox potential (mV), Cys-Cysteine, 3MH-3-Methylhistidine, Asp-Aspartate, Phe-Phenylanine, Trp-Tryptophan, Asn-Asparagine, Ala-Alanine, Pro-Proline, Erg-Ergothioneine, B.Ala-β-Alanine, GSH Redox –GSH/GSSG redox potential (mV), AdoHcy - S-adenosylhomocysteine, GSSG - GSH disulfide.

The correlation between plasma GSH and hepatic GVHD severity was determined using the paternal to F1 hybrid model (**Table 1**, model 2). This model was chosen because inter-animal variations in GSH and hepatic GVHD were greater than in model 1, and thus this model provided an opportunity to examine potential correlations between plasma metabolites and the severity of GVHD in the liver. As shown (**Figure 4**), histopathological scores and plasma GSSG were strongly and positively correlated (r^2^ = 0.65; p = 0.002). Significant positive correlations were also were observed in the GSH/GSSG redox potential (more positive = more oxidized). Significant inverse correlations were also observed between liver histopathological damage and plasma total cysteinylglycine (T. Cysgly; GSH catabolite), GSH, and total GSH (T. GSH), suggesting that concurrent plasma GSH/GSSG oxidation state reflects the extent of GVHD injury in the liver.

Several metabolites unrelated to GSH metabolism also were significantly correlated with histopathological changes in the liver (**Figure 4**). These metabolites include: (a) β-alanine, a degradation product of dipeptides, carnosine, anserine, and pantothenic acid (vitamin B5); (b) S-adenosylhomocysteine (AdoHcy), a product of S-adenosylmethionine (AdoMet) methylation; (c) Leucine/isoleucine, branched chain amino acids.

Interestingly, plasma Arg, a requisite precursor for NO synthesis, and Cit, also generated by iNOS enzymes, failed to correlate with liver histopathology. Plasma Trp, which is a substrate of IDO enzymes previously implicated in GVHD, also did not correlate with liver histopathology.

### Increased GSH Metabolic Precursors and Catabolites in Allo BMT Mice Suggest Both Impaired GSH Synthesis and Enhanced Turnover

**Figure 5:**
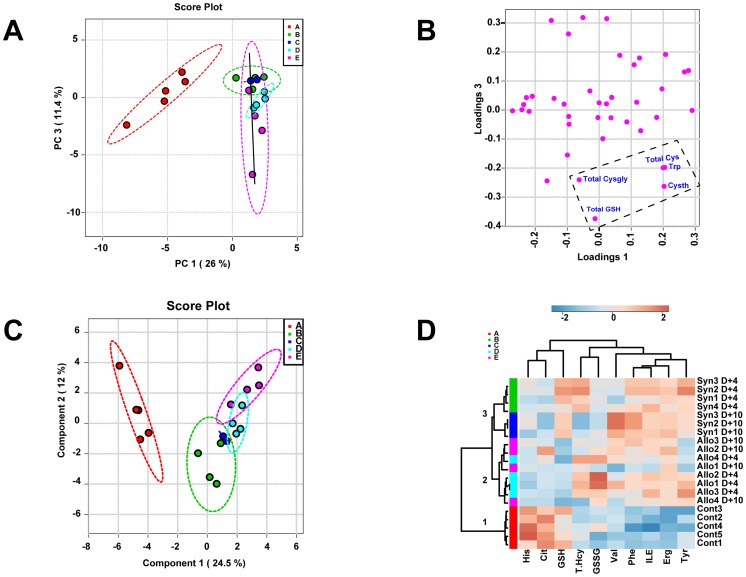
Plasma metabolome discriminates Allo from Syn BMT and untreated controls at Day+4. Lethally irradiated B6 recipients were transplanted with 5×10^6^ T-cell depleted bone marrow cells and 3×10^6^ CD90^+^ T-cells from B6 Thy1.1 (Syngeneic) or Balb/C (Allogeneic) donor mice (N = 4 per group). Principal component analysis (PCA) and partial-least squares discriminant analysis (PLS-DA) was performed using plasma metabolite concentrations quantified at Day+4. **Panel A** shows the PCA scores plot. The different colors and letters signify the five groups in the study: Healthy controls (A; red), Syn Day+4 (B; Green), Syn Day+10 (C; Blue), Allo Day+4 (D; Cyan), and Allo Day+10 (E; Purple). Untreated controls and the BMT groups are separated along the PC1 axis whereas Allo are separated from the Syn group along the PC 3 y-axis. Solid line shows the direction of Allo separation from Syn. **Panel B** shows the corresponding PCA loading plot for PC1 and PC3 shown in panel A. Total Cysgly, GSH, Cys, Trp and Cysth were variables that contributed the most to the separation of groups identified by the PCA analysis. **Panel C** shows the PLS-DA scores plot. The group IDs are represented by letters and colors described in Panel A. **Panel D** shows the heat-map generated from the top 10 metabolites contributing to group discrimination as identified by PLS-DA analysis. Each metabolite is arranged in columns and the individual concentrations within a column are normalized by respective median concentrations. Rows represent different mice and their group ID is shown on the right side of each row. These group IDs are represented by different colors on the left side that correspond to the same color codes in Panels A and C. Concentrations that are two fold above or below the mean are highlighted in amber or in blue, respectively. Dendogram and the 3 nodes (1–3) classified by hierarchical clustering analysis are shown on the left.

Supervised Principle Component Analysis (PCA) and unsupervised (PLS-DA) modeling permit identification of subtle metabolic shifts that may not achieve statistical significance in univariate analyses. These techniques were used to examine the metabolomic separation of the Allo and Syn BMT and baseline control groups. The PCA scores plot (**Figure 5**: Panel A) displays each mouse sample as a point on the plot (**Figure** **5**A) and shows the intrinsic segregation patterns of individual samples and group variances. An overview of the PCA score matrix using the first five principal components indicated that the best separation of Allo and Syn BMT mice was achieved when principal components 1 and 3 were used (data not shown). The first PC (PC 1, x-axis), which explained 26% of the variability in the data, separated the controls (A) from the other 4 groups: Syn BMT Day+4 (B) and +10 (C) and Allo BMT Day+4 (D) and +10 (E). The separation of Syn and Allo BMT groups at different time points is seen along the y-axis (PC 3), which is marked by the solid line (**Figure 5**: Panel A). As shown, Syn D+4 (B) and D+10 (C) had similar metabolomic compositions and closely overlapped with each other. Allo D+4 samples (D) segregated below the two Syn groups with a more significant separation being achieved with Allo D+10 (E) samples.

In the PC1 versus PC3 loading plot (**Figure 5**), Panel B illustrates that the plasma levels of Total Cys, Cysgly, GSH, Trp and Cysth were key segregating features that discriminated Allo D+10 from Syn groups. Accumulation of plasma GSH precursors (Cysth, Cys) and its catabolite (Cysgly) implicate impaired GSH synthesis in early GVHD. This is further supported by the GCL mRNA data shown in **Figure 2**. Although Total Cys, Cysgly, Trp and Cysth were not significantly different by univariate analysis, the PCA results suggest that these variables collectively discriminate between Allo and Syn groups, and demonstrate that early GVHD is associated with broad perturbations in sulfur amino acid metabolism in Allo mice.

Supervised PLS-DA analysis (**Figure** **5**C and D) was performed to confirm the class separation revealed by the PCA modeling. A five-class model was built to differentiate the five groups defined in Panel A: baseline controls (A), Syn (Day+4 (B), Day+10 (C)) and Allo (Day+4) (D), Day+10 (E)). As with results of the PCA analysis, segregation of the different BMT groups from the baseline controls occurred in the direction of Component 1, which explained 25% of the variance in the data set (**Figure** **5**C). Syn Day+4 and +10 showed overlapping metabolomic profiles and no separation was observed within the Syn group. Significant separation of Allo groups is evident in Day +4 and +10 groups with +10 groups showing a more profound separation from Syn groups. In PLS-DA analysis, both R^2^ and Q^2^ are used to test the predictive power of the model. A “Leave-One-Out” cross-validation test using a 3 component model gave an R^2^(Y) = 0.98 and Q^2^(cum) = 0.8, indicating that the PLS-DA model explains 98% of response variability. Q^2^ is an estimate of the predictive ability of the model and a value greater than 0.5 is regarded as good [42]. A permutation test with 2000 permutations and separation distance as a test statistic was significant (p = 0.025) and confirmed that this model had greater Q^2^ and R^2^ values than distributions calculated from permuted data.

To investigate additional group segregation, the top variables identified by PLS-DA analysis were subjected to hierarchical cluster analysis (HCA) to generate a heat-map shown in **Figure** **5**D. As shown, all the metabolites used in the HCA analysis had Variable Importance in Projection (VIP) values >1. The VIP is a computation of influence of every x term in the model on the group classification and larger VIP values indicate a greater influence of x on group discrimination; a VIP value of ≥ 1 is considered significant. In **Figure** **4**D, the rows represent each sample and the columns show the respective metabolite concentrations. The colors in each cell range from dark blue to brown and represent concentration changes from extreme low to high. The dendogram tree shown on the left (**Figure** **5**D) reveals three main clusters composed of baseline controls (Cluster 1; A), Allo (Cluster 2; D and E) and Syn (Cluster 3; B and C). Note that GSH and GSSG cells from Allo mice have a very distinct distribution compared to the Syn group. PLS-DA analysis also identified His, Cit and branched chain amino acids (Ile, Val), and aromatic amino acids (Phe, Tyr) but as shown in **Figure** **5**D, these variables were useful in discriminating the baseline controls from BMT recipients but not for segregating Allo from Syn BMT group.

**Figure 6:**
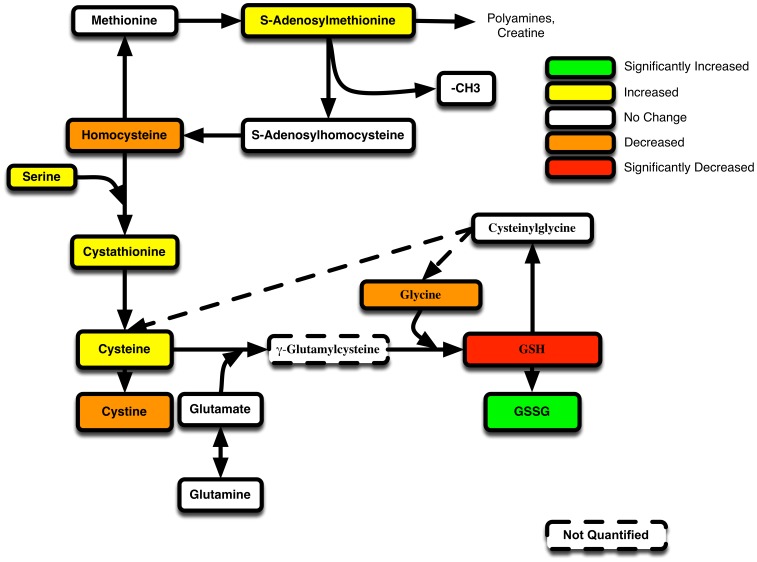
GVHD associated shifts in GSH metabolism in Allo relative to Syn BMT at Day+4. The pathway map of GSH-synthesis related metabolites are shown. The significance (p value) and the trends for the mean concentration differences between Allo and Syn mice are denoted by different colors. Green represents metabolites with mean concentrations in Allo mice that are significantly (p>0.5) increased (>25%) over syn mice. Yellow represents metabolites that are increased (>25%) in Allo, but was not statistically significant (p>0.05). White denotes metabolites that did not change greater ±25%. Orange represents metabolites whose mean concentration in Allo decreased by more than 25%, but did not reach significance (p>0.05). Red identifies metabolites that decreased in Allo by more than 25% and was also significant (p<0.05).

A summary of changes in the GSH metabolic pathway on Day+4 is presented in Figure **6**. The significant decline in plasma GSH and rise in GSSG is noted by red and green respectively. Despite low GSH in Allo mice, the concentrations of its rate-limiting substrate, Cys, trended upward (p = 0.3), suggesting it was not limiting. Intermediates (S-adenosylmethionine, Cysth, Ser) that lead to endogenous Cys production were all elevated in Allo mice, which suggests that Allo perturbations specifically impair GSH homeostasis without altering the upstream pathways that supply Cys required for its synthesis (**Figure 6**).

## Discussion

The major aims of the current work were to identify metabolic pathways and metabolites that are perturbed early in the course of developing GVHD after Allo BMT transplantation. Additionally, we aimed to identify plasma metabolic correlates of hepatic GVHD injury during the early clinical stages of GVHD. Using both conditioning-dependent and independent models of GVHD, rapid loss of plasma GSH and accumulation of its oxidized form occurs in early stages (Day +4) (**Tables 2**, **4**). These effects on the plasma GSH/GSSG redox state preceded the TNF-α induction that is associated with clinical GVHD (**Figure 3**), suggesting that oxidative stress is an upstream-event in the pathogenesis of GVHD. This was further confirmed by our data that showed early (Day +4) rise in hepatic oxidant production and protein carbonyl formation in Allo relative to Syn mice (Figure 2.). Despite significant increase in liver oxidative stress, cellular GSH synthesis enzyme expression was lower in Allo when compared to Syn group (Figure 2). Decreased hepatic GSH synthetic activity during early GVHD was further corroborated by our observation of lowered hepatic GSH and increased accumulation of its precursor metabolites in Allo animals (**Table 3** and **4**). Collectively, these data establish that a compensatory GSH antioxidant defense response observed in the Syn group (**Table 3** and **4**) is largely absent in Allo mice and is apparent very early in GVHD pathogenesis. Correlation analysis of hepatic histopathological scores with plasma metabolites at Day+10 in the paternal into F1 GVHD model also showed that the severity of hepatic injury is correlated with increased oxidation of plasma GSH. The implications of these results are discussed below.

### Sensitive Plasma and Hepatic GSH Depletion during Early GVHD

Comprehensive analysis of the major plasma amino acids and secondary metabolites of arginine catabolism allowed us to establish the relative sensitivity of plasma and hepatic GSH changes compared to other amino acid catabolic pathways. The panel of 40 analytes quantified included the 20 major amino acids, secondary metabolites produced from Arg catabolism and SAA-derived metabolites. Arg catabolism to citrulline and ornithine is increased during inflammation and we have shown previously in RAW macrophage cells that Arg loss and increased formation of its products are most sensitive biomarkers of macrophage activation [30]. Increases in urinary kynurenine which is synthesized from Trp by the enzyme indoleamine 2,3-dioxygenase (IDO) has been implicated as early biomarker of GVHD [43]. Despite their known involvement in GVHD, our data suggest that plasma and hepatic GSH and GSSG are more sensitive than Arg or Trp-related metabolites in detecting early hepatic oxidative stress injury.

To the best of our knowledge, acute changes in plasma and liver GSH metabolism following Allo and Syn BMT and during the early GVHD period have not been systematically examined. Sari and co-workers reported decreases in plasma antioxidant enzyme activities and increases in plasma lipid oxidation in Allo BMT patients 30 days post-HSCT [44]. Using a murine model of GVHD, Amer and co-workers reported an increased cellular oxidant burden and decreased GSH status in erythrocytes and in lymphocytes in GVHD mice 5 weeks following transplantation [28]. While results of these studies [3], [25], [28], [31], [44] are consistent with findings reported here, because these earlier studies do not focus on the early GVHD initiation period, it was unclear whether GSH depletion and oxidation are secondary consequences of inflammation or are preceding events that are involved in the initiation of alloreactivity. By comprehensively measuring GSH pathway metabolites and GSSG, our results establishes that depletion of GSH occurs prior to TNF-α induction and implicate them in the GVHD initiation process. Studies using a murine model of transplant associated Idiopathic Pneumonia Syndrome (IPS) [4], [27], [32], found that Allo BMT caused early depletion of lung and hepatic GSH. Because assessments of inflammation and histopathology were not performed in this report, the temporal relationship between GSH depletion, cytokine upregulation, and subsequent tissue damage was not established.

### Hepatic Oxidative Stress is an Early Event that Precede systemic Rise in TNF-α

Liver has one of the highest tissue concentrations of GSH. Plasma GSH concentrations are primarily determined by hepatic GSH biosynthesis and efflux [39], [45]. Hepatic GSH efflux into the plasma decreases proportionally when liver GSH declines [46]. GSSG efflux also increases in accordance with intrahepatic concentrations [47]. Oxidative stress increases liver GSSG, leading to its increased export through multi-drug resistant protein (MRP) transporters [48]–[50]. A previous study has shown that initial signal for T-cell infiltration can be detected as early as Day+3 following transplantation [51]. Consistent with this finding, evidence for significant rise in hepatic oxidative stress was obtained as early as Day +4 following Allo BMT. As presented in figure 2, hepatocytes isolated from Allo hosts at Day+4 exhibited a significantly higher ROS burden and accumulated more protein oxidation than those from Syn mice. GSSG concentrations in livers of Allo mice were significantly higher than livers of both baseline and Syn mice (**Figure 3** and **Table 2**). Thus, increased plasma GSSG may in part be due to increases in hepatic GSSG export. Protein carbonyl concentrations that are formed as a consequence of protein oxidation also increased in Allo BMT at Day+4 and clearly establish that liver oxidative stress and damage occurs prior to the rise in circulating TNF- α.

Despite an early increase in hepatic oxidative stress, mRNA abundance of GCLC, the rate-limiting enzyme in GSH synthesis, and total GSH were significantly lower in Allo mice in comparison to the Syn group. Total hepatic GSH concentrations in Syn mice were increased by ∼70% relative to baseline (**Table 4**), suggesting that transcriptional upregulation of the GSH antioxidant defense system may be responsible for decreased ROS in Syn hepatocytes. The mechanism underlying an Allo-specific dysregulation of the cellular antioxidant response remains unclear. It is possible that alloreactive T cells secrete cytokines or factors that dysregulate GSH homeostasis. Changes in localized cytokine levels within tissues such as the liver may not be detected in plasma, whereas, acute changes in liver GSH metabolism may be more sensitively reflected in the plasma compartment during early GVHD.

### Early GSH/GSSG Redox Dysregulation may Increase Severity of Inflammation and GVHD Through NFkB Dependent and Independent Mechanisms

Key findings in this study are that the GVHD-associated plasma GSH/GSSG ratio declines at Day+4 before TNF-α induction, and that plasma GSH depletion and oxidation at Day+10 correlates with hepatic GVHD severity. Though potential cause-and-effect relationships between early plasma GSH loss and subsequent cytokine elevation or GVHD severity were not addressed in this study, several precedents suggest such a causal relationship is plausible. (a) Host GSH/GSSG redox potential change may impact NFκB-dependent inflammatory signaling [52]–[54]. Cellular GSH depletion leads to sensitization of peroxide or reactive nitrogen species-dependent activation of NFκB [53], [54]. Although cellular mechanisms are incompletely understood, GSH depletion may potentiate NFκB activation through potentiating oxidation of tyrosine phosphatases such as Map Kinase Phosphatase-1 (MKP-1) [55] and Dynein light chain (LC8) [56]. (b) In addition to direct involvement in NFκB, accumulation of extracellular GSSG and protein oxidation may enhance Damage-Associated Molecular Pattern (DAMP)-mediated inflammatory signaling through S-glutathionylation of DAMP molecules such as high mobility group protein B1 (HMGB1) [57], [58]. (c) GSSG has been shown to enhance activities of cell surface leukocyte adhesion molecules, such as vascular adhesion molecule-1 (VCAM-1) and Intercellular adhesion molecule −1 (ICAM-1) [59]–[61] resulting in increased inflammation.

### Addressing the Impaired GSH Biosynthetic Pathway: A Therapeutic Target for GVHD Prevention?

These results emphasize the importance of understanding the mechanisms that lead to increased oxidative stress during GVHD pathogenesis, and also point to GSH biosynthesis as a potential target for GVHD prevention. How this might be accomplished depends on which component of the GSH homeostatic system is impaired in the early stages after transplantation. GSH is an endogenously synthesized compound, which is poorly taken up through diet. Clinical approaches to raise GSH have predominantly been focused on N-acetylcysteine (NAC) as a cysteine pro-drug to boost cellular GSH synthesis. However, the metabolic data presented here indicate that loss of GSH during early GVHD is not due to limited cysteine availability, and therefore suggest that such an approach might not be efficacious. Consistent with this idea, a clinical intervention with a moderate dose (100 mg/kg per day) of NAC increased rather than decreased the prevalence of GVHD and veno-occulusive disease of the liver (VOD) in Allo BMT patients [62], [63]. Another contributing factor in the failure of NAC to prevent GVHD is that NAC may exert non-GSH dependent immune-stimulating effects on Allo T cells [62]. Evidence presented in this paper suggests that an alternative intervention aimed at increasing cellular GSH synthesis capacity may be effective in relieving the initial oxidative stress associated with GVHD.

## Conclusion

This report utilizes a metabolomics approach to demonstrate that oxidative stress driven by impaired hepatic GSH biosynthesis occurs early in GVHD and precedes cytokine upregulation is the hallmark of the onset of GVHD. Results help to clarify the mechanisms resulting in oxidative stress during early GVHD. They also identify hepatic ROS generation and the GSH synthesis pathway as potential targets for early intervention. Plasma GSH and GSSG may also be clinically useful biomarkers for early GVHD prediction. Future work will be directed at investigating upstream events following transplantation that culminate in disruption of the GSH biosynthetic pathway.
